# Correlation of serum levels of fibroblast growth factor 23 and Klotho protein levels with bone mineral density in maintenance hemodialysis patients

**DOI:** 10.1186/s40001-018-0315-z

**Published:** 2018-04-17

**Authors:** Shubei Zheng, Yan Chen, Yu Zheng, Zhihong Zhou, Zhanyuan Li

**Affiliations:** Department of Nephrology, The Second Affiliated Hospital & Yuying Children’s Hospital of Wenzhou Medical University, 109 Xueyuanxi Rd., Wenzhou, 325027 People’s Republic of China

**Keywords:** Hemodialysis, Bone mineral density, Fibroblast growth factor 23, Klotho protein

## Abstract

**Objective:**

The correlation of serum fibroblast growth factor 23 (FGF-23) and Klotho protein levels with bone mineral density (BMD) in maintenance hemodialysis (MHD) patients was analyzed.

**Methods:**

Between January 2015 and November 2015, 125 MHD patients in our hospital were enrolled. Dual-energy X-ray absorptiometry was used to examine the BMD in the femoral neck and lumbar spine of MHD patients. The patients were divided into three groups: a normal bone mass group, an osteopenia group, and an osteoporosis group. An ELISA was performed to measure serum FGF-23, Klotho protein, and 1,25(OH)_2_VitD_3_ levels. Other parameters, including calcium (Ca), phosphorus (P), and parathyroid hormone, were also measured.

**Results:**

Of the 125 MHD patients, 82.40% of patients had femoral neck osteopenia, and 56.00% of patients had lumbar spinal osteopenia. The serum FGF-23 level was highest in the osteoporosis group. However, there was no significant difference in serum FGF-23 levels among the three groups, depending on femoral neck and lumbar spinal BMD (*P* > 0.05). Spearman’s correlation analysis also pointed to a lack of correlation between serum FGF-23 levels and BMD. Among the three groups, there were significant differences in serum Klotho protein levels and femoral neck BMD (*P* < 0.05). Serum Klotho protein levels in the osteoporosis group were clearly lower than those in the normal bone mass group and osteopenia group (*P *< 0.05). Similarly, serum Klotho protein levels were significantly lower in those with lumbar spinal osteopenia as compared with those in the normal group. There was a positive correlation between serum Klotho protein levels and BMD and T values for the femoral neck and lumbar spine. The results of a multiple linear regression analysis revealed that the serum Klotho protein level was one of the main factors affecting BMD in MHD patients.

**Conclusions:**

The serum level of FGF-23 was not correlated with a change in BMD of MHD patients, whereas the serum Klotho protein level was associated with the degree of BMD. A high Klotho protein level may decrease the severity of chronic kidney disease and mineral bone disorder (CKD-MBD) in MHD patients with low BMD.

## Background

In end-stage renal disease, patients require maintenance hemodialysis (MHD) to improve their quality of life and extend their lives [1, 2]. Due to a decline in mineral substances, especially calcium (Ca) and phosphorus (P), chronic kidney disease and bone mineral disorder (CKD-MBD), together with low bone mineral density (BMD), is common in MHD patients [3]. Fibroblast growth factor 23 (FGF-23) plays a key role in regulating P in the human body and therefore in bone metabolism of MHD patients [4]. The Klotho protein is a cofactor, which induces activation of the FGF-23 receptor to produce biological effects of FGF-23 [5]. Mice lacking the Klotho protein exhibited severe osteoporosis, depleted numbers of osteoblasts, decreased alkaline phosphatase activities, potentially weakened bone formation, and a large nonmineral region in trabeculae and metaphysis of bone [6].

The present study examined the correlation between serum levels of FGF-23 and serum Klotho protein levels with BMD in MHD patients to determine the influence of the FGF-23/Klotho axis on CKD-MBD, accompanied by low BMD.

## Patients and methods

### Patients

The study consisted of 125 patients who underwent MHD between January 2015 and November 2015 at the Second Affiliated Hospital and Yuying Children’s Hospital of Wenzhou Medical University. The patient group comprised 73 males and 52 females, ranging in age ranging from 30 to 89 years. The average age was 63.35 ± 14.69 years. The duration of hemodialysis ranged from 18 to 213 months, and the median duration of hemodialysis was 65.00 (52.00, 87.50) months. Among the 125 MHD patents, 45 (36.00%) patients had chronic glomerulonephritis, 31 (24.8%) patients had hypertensive kidney lesions, 29 (23.20%) patients had diabetic nephropathy, 10 (8.00%) patients had polycystic kidney disease, and 10 (8%) patients had other conditions. Patients with any of the following were excluded: symptoms of autoimmune diseases; malignant tumors, active hepatitis, cirrhosis, thyroid disease, acute infections, severe malnutrition, or a mental disorder or other disease that precluded examinations. In addition, those with a history of hormone therapy or immunosuppressive therapy in the past 6 months, a history of excision of parathyroid glands, or a history of thoracolumbar and femoral fractures in the past 6 months were excluded.

In all cases, MHD was performed with bicarbonate dialysate containing 1.5 mmol/L of Ca for 4–5 h three times each week using a POLYFLUX 14L dialyzer. For vascular access, a native arteriovenous fistula or long-term internal jugular vein cannulation was employed. The blood flow was 200–250 mL/min. The dialysate flow rate was 500 mL/min. At each hemodialysis session, the ultrafiltration volume of dialysis was estimated by a clinically derived value of dry weight.

This study was approved by the ethics committee of the Second Affiliated Hospital and Yuying Children’s Hospital of Wenzhou Medical University, and written informed consent was obtained from all the patients.

### General clinical data

The clinical data of all patients, including age, gender, protopathy, hemodialysis duration, body mass index (BMI), smoking history (more than ten cigarettes every day for more than 5 years), and diabetes and high blood pressure histories, were collected and analyzed.

### Measurement of BMD

DXA-MD dual-energy X-ray absorptiometry (Lunar Prodigy; General Electric, CA, USA) was used to measure BMD in the posteroanterior lumbar spine (L1–L4) and femoral neck. BMD was represented as an absolute value of the BMD and *T* score. The *T* score was determined as follows: (the measured value − the peak bone mass)/standard deviations of BMD of a healthy adult. According to the diagnostic criteria of the World Health Organization, the patients were divided into the following three groups: a normal bone mass group (T > − 1.0), an osteopenia group (− 2.5 < T ≤ − 1.0), and an osteoporosis group (T ≤ − 2.5),

### Measurement of hematological parameters

Fasting venous blood samples were collected before dialysis 1 month after the day of the BMD examination. Hemoglobin, albumin, blood glucose, Ca, P, and parathyroid hormone (PTH) were determined. Ca, Ca-P product (Ca × P), and the urea clearance index were adjusted. The levels of FGF-23, Klotho protein, and 1,25(OH)_2_VitD_3_ in human serum were detected by an ELISA kits (Bio-Swamp Company, NY, USA), according to the manufacturer’s instructions.

### Statistical analysis

Statistical analysis of the data was performed using SPSS 19.0 software. Normally distributed measurement data were presented as *x* ± SD. The difference was compared with an analysis of variance and the LSD method. Measurement data with an abnormal distribution are presented as M (1/4, 3/4), the difference of which was compared with the Kruskal–Wallis H test and Mann–Whitney *U* test, and adjusted by the Bonferroni test. Enumeration data are presented as case number and percentage. The difference was compared with a Chi-square test and Bonferroni test. Spearman’s correlation was conducted to analyze the relation of serum levels of FGF-23, Klotho protein levels, and other parameters with changes in BMD. A multiple linear regression analysis was performed to determine the factors associated with a change in the BMD of MHD patients. A *P* value less than 0.05 denoted a statistically significant difference.

## Results

### Comparison of clinical indices

Among the 125 MHD patients, the BMD measurements of the femoral neck revealed 62 (49.60%) cases of osteoporosis and 41 (32.80%) cases of osteopenia. In 22 (17.60%) cases, the bone mass was normal. The measurements of the BMD of the lumbar spine revealed 34 (27.20%) cases of osteoporosis and 36 (28.80%) cases of osteopenia. The bone mass was normal in 55 (44.00%) patients. In terms of age, patients with femoral neck osteoporosis were older than those with femoral neck osteopenia and those with normal bone mass (*P* < 0.05). Patients with femoral neck osteoporosis also showed a significant decline in serum Klotho protein levels. When the patient’s age and serum Klotho protein level of the osteopenia group with normal bone mass in the femoral neck were compared, there was no significant difference. The adjusted levels of Ca, P, Ca × P, PTH, and 1,25(OH)_2_VitD_3_ and FGF-23 showed no significant difference among the three groups (Table 1).

**Table 1:** Comparison of index among divided groups according to BMD of femur neck

Item	Normal bone mass (*n* = 22)	Osteopenia (*n* = 41)	Osteoporosis (*n* = 62)	*P* value
Age (years)	56.091 ± 15.832	59.976 ± 14.828	68.161 ± 12.575^a,b^	0.001
Male [case (%)]	14 (63.64)	29 (70.73)	30 (48.39)	0.068
Smoking [case (%)]	6 (27.27)	10 (24.39)	18 (29.03)	0.874
Diabetes [case (%)]	6 (27.27)	11 (26.83)	29 (46.77)	0.072
Hypertension [case (%)]	14 (63.64)	29 (70.73)	46 (74.19)	0.641
Hemodialysis duration [month, M (1/4, 3/4)]	64.50 (51.00, 70.25)	59.00 (52.00, 85.00)	68.00 (54.00, 93.25)	0.680
Kt/*V*	1.752 ± 0.299	1.831 ± 0.489	1.859 ± 0.462	0.628
BMI (kg/m^2^)	21.448 ± 3.651	21.244 ± 3.695	20.224 ± 2.839	0.178
Hb (g/L)	105.500 ± 11.300	105.390 ± 12.438	104.726 ± 11.857	0.947
Glu [mmol/L, M (1/4, 3/4)]	6.70 (5.69, 7.90)	7.05 (5.71, 8.71)	7.15 (5.83, 10.03)	0.412
Alb (g/L)	40.032 ± 1.845	39.585 ± 3.190	39.076 ± 2.569	0.318
Adjusted Ca (mmol/L)	2.237 ± 0.258	2.314 ± 0.185	2.304 ± 0.193	0.321
P (mmol/L)	1.501 ± 0.450	1.536 ± 0.466	1.666 ± 0.457	0.217
Ca × P (mg^2^/dL^2^)	40.397 ± 13.849	44.991 ± 13.702	47.710 ± 14.122	0.107
PTH [ng/L, M (1/4, 3/4)]	225.70 (104.40, 750.68)	290.90 (131.30, 815.70)	432.50 (217.78, 889.63)	0.132
1,25(OH)_2_VitD_3_ (ng/mL)	32.486 ± 6.482	33.095 ± 6.080	32.612 ± 6.756	0.914
FGF23 (ng/L)	99.639 ± 22.052	92.646 ± 19.382	102.013 ± 20.085	0.072
Klotho protein (ng/L)	429.883 ± 41.776	410.598 ± 61.056	387.172 ± 54.137^a,b^	0.005

^a^*P* < 0.05 vs Normal bone mass

^b^*P* < 0.05 vs Osteopenia

The proportion of males was markedly lower in the lumbar vertebral osteoporosis group than in the normal bone mass lumbar vertebral group, and there was no significant difference with the osteopenia group (*P *< 0.01). As compared with the osteopenia group with a normal bone mass in the lumbar vertebrae, the BMI of the group with osteoporosis in the lumbar vertebrae was significantly decreased (*P *< 0.05). The comparison of the BMI between the osteopenia group with normal bone mass in the lumbar vertebrae showed no significant difference. There was a significant difference in serum Klotho protein levels in the three groups. The serum Klotho protein level in the group with lumbar vertebral osteopenia was clearly lower than that of the group with a normal bone mass in the lumbar vertebrae. There was no significant difference in the group with lumbar vertebral osteoporosis and the other two groups. The adjusted levels of Ca, P, Ca × P, PTH and 1,25(OH)_2_VitD_3_, and FGF-23 showed no significant difference among the three groups (Table 2).

**Table 2:** Comparison of index among divided groups according to BMD of lumbar spine

Item	Normal bone mass (*n* = 55)	Osteopenia (*n* = 36)	Osteoporosis (*n* = 34)	*P* value
Age (years)	61.346 ± 16.304	66.111 ± 13.846	63.677 ± 12.557	0.317
Male [case (%)]	40 (72.73)	21 (58.33)	12 (35.29)^b^	0.002
Smoking [case (%)]	19 (34.55)	9 (25.00)	6 (17.65)	0.207
Diabetes [case (%)]	19 (34.55)	15 (41.67)	12 (35.29)	0.771
Hypertension [case (%)]	38 (69.09)	28 (77.78)	23 (67.65)	0.580
Hemodialysis duration [month, M (1/4, 3/4)]	61.00 (47.00, 87.50)	56.00 (46.00, 64.50)	63.00 (49.25, 90.50)	0.194
Kt/*V*	1.915 ± 0.492	1.708 ± 0.255	1.825 ± 0.505	0.095
BMI (kg/m^2^)	21.235 ± 3.358	21.330 ± 3.599	19.440 ± 2.523^a,c^	0.021
Hb (g/L)	106.382 ± 12.458	102.639 ± 10.944	105.559 ± 11.771	0.329
Glu [mmol/L, M (1/4, 3/4)]	6.81 (5.64, 9.07)	7.56 (5.82, 9.84)	7.26 (5.84, 9.52)	0.499
Alb (g/L)	39.762 ± 2.826	39.256 ± 2.560	39.009 ± 2.605	0.407
Adjusted Ca (mmol/L)	2.311 ± 0.236	2.287 ± 0.192	2.280 ± 0.160	0.749
P (mmol/L)	1.552 ± 0.461	1.591 ± 0.412	1.667 ± 0.511	0.522
Ca × P (mg^2^/dL^2^)	45.106 ± 13.833	44.893 ± 13.307	46.894 ± 15.541	0.804
PTH [ng/L, M (1/4, 3/4)]	328.40 (125.05, 604.10)	349.00 (182.93, 887.40)	744.00 (225.18, 1129.25)	0.062
1,25(OH)_2_VitD_3_ (ng/mL)	32.376 ± 5.923	34.585 ± 7.298	31.405 ± 6.039	0.101
FGF23 (ng/L)	93.930 ± 20.350	101.437 ± 19.277	102.865 ± 21.012	0.080
Klotho protein (ng/L)	417.108 ± 56.179	387.263 ± 53.255^a^	394.534 ± 56.770	0.030

^a^*P* < 0.05 vs Normal bone mass

^b^*P* < 0.01 vs Normal bone mass

^c^*P* < 0.05 vs Osteopenia

### Correlation between BMD and clinical indices

The results of Spearman’s correlation analysis indicated that the BMD in the femoral neck showed a positive correlation with the Klotho protein level and a negative correlation with the patient’s age and serum P level. The T value for the femoral neck showed a positive correlation with the Klotho protein level and a negative correlation with patient age, P, Ca × P, and PTH. Both the BMD and *T* value for the lumbar vertebrae revealed a positive correlation with the level of the Klotho protein and BMI and a negative correlation with PTH (Table 3). The results of Spearman’s correlation analysis revealed no correlation between BMD and serum levels of FGF-23.

**Table 3:** Spearman correlation analysis between the BMD of 125 MHD patients and clinical index

Variable	BMD of femur neck		*T* score of femur neck		BMD of lumbar spine		T score of lumbar spine	
	*r*	*P* value	*r*	*P* value	*r*	*P* value	*r*	*P* value
Age	− 0.324	0.000	− 0.345	0.000	− 0.020	0.824	− 0.026	0.770
BMI	0.128	0.156	0.162	0.071	0.180	0.045	0.194	0.030
Serum P	− 0.185	0.039	− 0.200	0.026	− 0.065	0.472	− 0.067	0.458
Ca × P	− 0.167	0.063	− 0.182	0.042	0.015	0.867	0.017	0.853
PTH	− 0.161	0.072	− 0.189	0.035	− 0.233	0.009	− 0.243	0.006
Klotho protein	0.252	0.005	0.297	0.001	0.202	0.024	0.202	0.024

### Factors affecting BMD

Treating PTH with a natural logarithm to follow a normal distribution, as the independent variable, the age, BMI, P, lnPTH, and Klotho protein were input into a multiple regression analysis. The results indicated that an increased serum Klotho protein level could predict CKD-MBD and a low BMD in MHD patients. Older age was an independent risk factor for reduced BMD in the femoral neck in MHD patients. BMI was an independent risk factor for decreased lumbar vertebral BMD (Table 4).

**Table 4:** Multiple linear regression analytical method analyzed the factors of effect on the change of BMD in patients with MHD

	Variable	*β*	SE	*β*′	*t*	*P* value
Dependent variable: BMD of femur neck	Age	− 0.004	0.001	− 0.321	− 3.832	0.000
	Klotho protein	0.001	0.000	0.192	2.285	0.024
Dependent variable: *T* score of femur neck	Age	− 0.032	0.007	− 0.379	− 4.563	0.000
	Klotho protein	0.004	0.002	0.186	2.281	0.024
Dependent variable: BMD of lumbar spine	BMI	0.017	0.006	0.241	2.803	0.006
	Klotho protein	0.001	0.000	0.184	2.144	0.034
Dependent variable: *T* score of lumbar spine	BMI	0.144	0.049	0.251	2.940	0.004
	Klotho protein	0.006	0.003	0.186	2.172	0.032

## Discussion

Patients with MHD commonly suffer from CKD-MBD and low BMD [3], which causes skeleton and joint pain. They also have an increased risk of fracture, disability, and mortality. Among the 125 MHD patients in whom BMD was evaluated by dual-energy X-ray absorptiometry, femoral neck BMD was decreased in 82.4% of patients, and lumbar vertebral BMD was reduced in 56% of cases, indicating that MHD patients have a high risk of decreased BMD.

FGF-23, which regulates P and vitamin D, is produced and secreted by bone cells and fibroblasts, and it plays an important role in bone metabolism of CKD patients. On the one hand, FGF-23 reduces the reabsorption of P from urine and enhances the excretion of urine P through downregulation of the Na+/phosphate cotransporter (Na/Pi-2a, Na/Pi-2c) in epithelial cells of renal proximal tubules. On the other hand, FGF-23 decreases the absorption of P in the small intestine by downregulating 1-hydroxylase to decrease 1,25(OH)_2_VitD_3_ and upregulating 24-hydroxylase to degrade 1,25(OH)_2_VitD_3_ to VD3-23-calcitroic acid [7]. In early-stage CKD, the FGF-23 level is elevated to maintain serum phosphate in the normal range through increased excretion of urine P and decreased absorption of P in the intestine. With deterioration of renal function, the level of FGF-23 continues to rise. In end-stage CKD, due to kidney tubule damage, the ability of FGF-23 to regulate P declines, and subsequent increases in P levels induce further rises in the FGF-23 level [7, 8]. Following the decline in the acid-secreting function of the kidneys, metabolic acidosis in bone cells results in further increases in the level of FGF-23. Krieger [9] reported that the expression of FGF-23 at the mRNA level was enhanced and that the serum concentration of FGF-23 was increased in mice with renal function damage and metabolic acidosis. Other studies demonstrated that increases in the level of FGF-23 not only reduced the level of 1,25(OH)_2_VitD_3_ but also inhibited the level of PTH and the regulation of estrogen, which is associated with bone tissue growth and development [10, 11]. Lane [12] showed that an increased level of serum FGF-23 enhanced the risk of fractures in patients with a glomerular filtration rate less than 60 mL min^−1^ (1.73 m^2^)^−1^, which suggested that the level of FGF-23 was correlated with a change in BMD in renal disease patients. Wang [13] reported that overexpression of FGF-23 not only inhibited the differentiation of bone matrix but also mineralization of bone matrix and suggested that overexpression of FGF-23 inhibited bone formation. However, Urena [14] showed that the level of FGF-23 in MHD patients was not correlated with BMD. Coskun [15] also found no relationship between serum FGF-23 levels and BMD values in bone diseases. In the present study, the MHD patients were divided into three groups (normal bone mass, osteopenia, and osteoporosis) according to BMD in the femoral neck and lumbar vertebrae. The results showed that FGF-23 levels were highest in the osteoporosis group, but there was no significant difference in FGF-23 levels among the three groups. Spearman’s correlation analysis confirmed the lack of correlation between BMD and FGF-23 levels. According to previous studies, the biological function of FGF-23 is absent in the presence of a decrease in Klotho protein levels [16, 17]. Hence, in the present study, we inferred that the Klotho protein level influenced the regulatory function of FGF-23 in bone formation.

The Klotho protein is a transmembrane protein encoded by the *Klotho* gene. Previous research reported that mice lacking the *Klotho* gene developed osteoporosis [18]. Transmission electron microscope observations showed that osteoblasts in bone trabecula in the metaphysis of tibias and femurs were diffusively distributed in mice with genetic defects of Klotho [19]. Moreover, organelles were less developed in osteoblasts, bone matrix without mineralization was increased, and calcium P precipitation was diffusively and unevenly distributed. The authors concluded that a lack of the Klotho protein not only decreased the number of osteoblasts but also influenced the function of osteoblasts, thereby interfering with bone mineralization. In the present study, serum Klotho protein levels in the patients with osteoporosis in the femoral neck were lower than those of the group with osteopenia and normal bone mass in the femoral neck. There was no significant difference in serum Klotho protein levels among the three groups when the patients were divided according to BMD values for the lumbar vertebra, although the serum Klotho protein level was clearly lower in the osteopenia group than that in the normal bone mass group. In addition to its role in the regulation of VD via the signal transduction of FGF-23, the Klotho protein also participates in the regulation of bone mineralization [20, 21]. It exerts its effects by regulating Ca homeostasis and adjusting Ca absorption in distal convoluted tubules, thereby stabilizing the activity of transient receptor potential vanilloid receptor 5 in the cytomembrane [20, 21]. Furthermore, the Klotho protein regulates the secretion of PTH to promote reabsorption of urinary Ca by facilitating Na–K-ATPase collection and dissemination to the apical membrane of chief cells of the parathyroid gland [22]. In addition, the Klotho protein functions as a kind of hormone, inhibiting signal transduction of insulin and insulin-like growth factor and regulating estrogen secretion [17, 23]. The protein also plays a role in combating the release of activated oxygen species in the body [24].

CKD-MBD, which is accompanied by low BMD in MHD patients, is a complex and multifactorial clinical disease. In the present study, older age was negatively correlated with a change in BMD in the femoral neck in MHD patients, and advanced age was an independent risk factor for low BMD in the femoral neck in patients with CKD-MBD. The BMI of patients with lumbar vertebral osteoporosis was obviously declined, and it was positively correlated with a change in BMD. Although serum phosphate, Ca × P, and PTH showed a negative correlation with a change in BMD values, these indexes were not significantly associated with a change in BMD in between-group comparisons of the groups. The use of Ca, P, and a P modifier may have activated VD during MHD. In future work, we will divide the groups according to the patients’ therapeutic regimens.

In summary, the present study suggested that the level of serum FGF-23 was not correlated with changes in BMD. Serum Klotho protein levels in MHD patients with osteoporosis were lower than those of MHD patients without osteoporosis, and these levels were positively correlated with changes in BMD. An increase in serum Klotho protein levels may decrease the risk of CKD-MBD and low BMD in patients with end-stage renal diseases. As this was a single-center cross-sectional study and the sample size was relatively small, we did not include data on the therapeutic regimen in the analysis. In addition, we did not determine the bone metabolic index due to conditionality and lack of follow-up data. Further studies are needed to explore the effect of the FGF-23/Klotho protein axis on MHD patients with low BMD.
